# Twenty-Four-Month Real-World Outcomes of Ofatumumab in Relapsing–Remitting Multiple Sclerosis: A Multicenter Retrospective Cohort Study

**DOI:** 10.3390/jcm15072585

**Published:** 2026-03-27

**Authors:** Weronika Galus, Magdalena Kiełbowicz-Hołysz, Joanna Siuda, Gabriela Gajewska, Anetta Lasek-Bal, Przemysław Puz

**Affiliations:** 1 Department of Neurology, Faculty of Medical Sciences in Katowice, Medical University of Silesia, 40-752 Katowice, Poland; jsiuda@sum.edu.pl; 2 Department of Neurology with the Stroke Subunit Prof. K. Gibiński University Clinical Centre, Medical University of Silesia in Katowice, 40-752 Katowice, Poland; magdalena.kielbowicz1506@gmail.com; 3 Department of Neurology, Upper Silesian Medical Center, Medical University of Silesia, 40-635 Katowice, Poland; ggajewska@gcm.pl; 4 Department of Neurology, Faculty of Health Sciences in Katowice, Medical University of Silesia, 40-635 Katowice, Poland; abal@sum.edu.pl (A.L.-B.); ppuz@sum.edu.pl (P.P.)

**Keywords:** multiple sclerosis, disease-modifying therapy, ofatumumab, real-world data

## Abstract

**Background/Objectives**: Real-world evidence on ofatumumab (OFA) beyond 12 months remains limited in relapsing–remitting multiple sclerosis (RRMS). We assessed 24-month effectiveness and safety, compared treatment-naïve and previously treated patients, and explored predictors of failure to achieve No Evidence of Disease Activity-3 (NEDA-3). **Methods**: This multicenter retrospective cohort study included adult RRMS patients treated with OFA in routine clinical practice. Effectiveness analyses were restricted to patients with complete 24-month follow-up and full clinical and magnetic resonance imaging (MRI) assessment (complete-case analysis). Outcomes included relapses, MRI activity, Expanded Disability Status Scale (EDSS) progression, NEDA-3, and adverse events (AEs). Exploratory multivariable logistic regression was used to assess baseline predictors of NEDA-3 non-achievement. **Results**: Of 258 patients who initiated OFA, 148 had completed 24-month clinical and MRI follow-up and were evaluable for effectiveness. Over 24 months, 71.5% achieved NEDA-3; relapses occurred in 15.5% of patients, MRI activity in 15.5%, gadolinium-enhancing lesions (GELs) in 4.7%, and EDSS progression in 17.6%. Disease activity was minimal during months 12–24, with relapses in 2.7%, MRI activity in 2.0%, and no GELs. In unadjusted analyses, no statistically significant differences were observed between treatment-naïve and previously treated patients. Higher baseline EDSS was associated with failure to achieve NEDA-3. In the 24-month safety subgroup, AEs were recorded in 28.4% of patients; infections occurred in 26.4% of patients (all grade 1–2), and no serious adverse events were observed. **Conclusions**: In this multicenter real-world cohort, OFA was associated with low inflammatory disease activity over 24 months in RRMS patients with complete follow-up. These findings should be interpreted cautiously because the effectiveness analysis was restricted to a complete-case cohort and safety data were collected retrospectively.

## 1. Introduction

Multiple sclerosis (MS) is a chronic immune-mediated disorder of the central nervous system in which inflammation and neurodegeneration lead to relapses, neurological disability, and reduced quality of life [1]. Although many disease-modifying therapies (DMTs) reduce relapse rates, a key unmet need in routine practice is durable suppression of inflammatory activity with a regimen that patients can maintain over years [2]. Real-world evidence (RWE) complements randomized trials by showing effectiveness, safety, and treatment persistence in routine care, across diverse patients and with less controlled monitoring [3].

B cells drive MS via antigen presentation and cytokines, shaping immune responses [4]; this has boosted anti-CD20 (cluster of differentiation 20) therapies as high-efficacy treatments for relapsing–remitting MS (RRMS). Ofatumumab (OFA) is a fully human anti-CD20 monoclonal antibody administered subcutaneously once monthly after an initial loading schedule, allowing home-based self-injection and potentially supporting treatment adherence [5]. In clinical and open-label extension trials, OFA reduced relapse activity and magnetic resonance imaging (MRI) inflammation compared with an active comparator, while maintaining an acceptable safety profile [6]. However, the translation of trial efficacy into routine practice depends on local eligibility rules, monitoring intensity, and patient selection within reimbursement programs.

In our previous multicenter analysis, we reported the first 12 months of OFA treatment in Polish tertiary referral centers and observed high disease control with a favorable tolerability profile in routine care [7].

This study extends follow-up to 24 months, assessing sustained clinical and radiological outcomes, differences between treatment-naïve and switched patients, and predictors of residual activity in a complete-case two-year cohort.

## 2. Materials and Methods

### 2.1. Study Design

The study design, patient eligibility, data sources, outcome definitions, safety grading, and statistical methods were consistent with our previous publication [7]. In brief, this retrospective cohort study included adult RRMS patients treated with OFA within the Polish Ministry of Health Therapeutic Program in tertiary MS centers [8]. Follow-up was conducted according to program standards, with regular neurological visits with Expanded Disability Status Scale (EDSS) assessment [9], routine laboratory monitoring, and annual contrast-enhanced brain MRI. EDSS assessments were performed by treating neurologists during routine visits at participating centers; because of the retrospective multicenter design, no central EDSS adjudication was available. Confirmed disability progression was defined as an increase of ≥1.0 EDSS point when baseline EDSS was ≤5.5 or ≥0.5 point when baseline EDSS was >5.5, confirmed after at least 6 months. Relapses were defined as new or worsening neurological events lasting ≥24 h, occurring without fever/infection and ≥30 days after a prior relapse. MRI activity comprised new or enlarging T2-weighted (T2-W) lesions and/or gadolinium-enhancing lesions (GELs). No Evidence of Disease Activity–3 (NEDA-3), defined as no relapses, no confirmed EDSS progression, and no MRI activity, was assessed. Because NEDA-3 required a complete 24-month clinical and MRI assessment, effectiveness analyses were restricted to patients who completed 24 months of OFA and had full clinical and radiological data available (complete-case analysis). Patients whose follow-up was still immature at database lock or who discontinued earlier were not evaluable for the 24-month effectiveness endpoint. Multivariable logistic regression was used as an exploratory analysis to assess baseline predictors of not achieving NEDA-3, including age, sex, disease duration, treatment status (treatment-naïve vs. switched), prior high-efficacy therapy (HET), and baseline EDSS. Safety analyses reported in this manuscript refer to the subgroup with mature follow-up and available longitudinal documentation, because retrospective adverse-event abstraction was not uniformly complete in patients whose follow-up was still immature at database lock.

### 2.2. Institutional Review Board Statement

The study complied with the Declaration of Helsinki and was approved by the Ethics Committee of the Medical University of Silesia (BNW/NWN/0052/KB1/141/I/22/23/24; 30 January 2024); written informed consent was waived.

### 2.3. Statistical Analysis

Continuous data are presented as mean ± standard deviation (SD) or median with interquartile range (IQR), and categorical data as counts and percentages. Groups were compared with *t*-test or Mann–Whitney U (continuous) and χ^2^ or Fisher’s exact test (categorical), as appropriate. Within-patient changes across three 12-month periods (pre-treatment, 0–12, and 12–24 months) in relapses and MRI activity were tested with Cochran’s Q; significant results were followed by McNemar pairwise tests with Holm correction. Baseline predictors of failing to reach NEDA-3 at 24 months were assessed using multivariable logistic regression, including age, sex, disease duration, treatment-naïve status, prior high-efficacy therapy exposure, and baseline EDSS. Because of the limited number of outcome events, this analysis was considered exploratory. Model discrimination was evaluated using the area under the receiver operating characteristic curve (AUC) with 95% confidence intervals. Analyses used STATISTICA 13.0 PL (Tibco, Palo Alto, CA, USA).

## 3. Results

### 3.1. Study Group Characteristics

At treatment initiation, 258 patients (188 women and 70 men) were included; mean age was 38.91 ± 11.28 years, mean disease duration was 9.30 ± 7.35 years, mean prior treatment duration was 5.59 ± 4.80 years, and mean EDSS was 2.14 ± 1.09 (median 2.0; IQR 1.5–2.5). Overall, 169 patients switched from another DMT, and 89 were treatment-naïve. Of these 258 patients, 148 completed 24 months of follow-up with complete clinical and MRI assessment and formed the 24-month effectiveness cohort (92 switched patients and 56 treatment-naïve patients). Baseline characteristics of this subcohort with mature follow-up are summarized in Table 1.

Of the 258 patients treated with OFA, 148 completed 24 months of follow-up and underwent a complete clinical and MRI assessment (56 treatment-naïve patients and 92 patients who switched from another DMT).

Most patients had prior exposure to platform therapies (n = 142), whereas the remaining patients had received HET (n = 27). Among patients who switched, the main documented reasons for switching to OFA were disease activity (n = 57; 61.9%), adverse events (n = 19; 20.7%), and the treating physician’s assessment that the switch would provide a therapeutic benefit to the patient (n = 16; 17.4%). Notably, this category corresponds to one of the formal criteria of the drug program.

**Figure 1:**
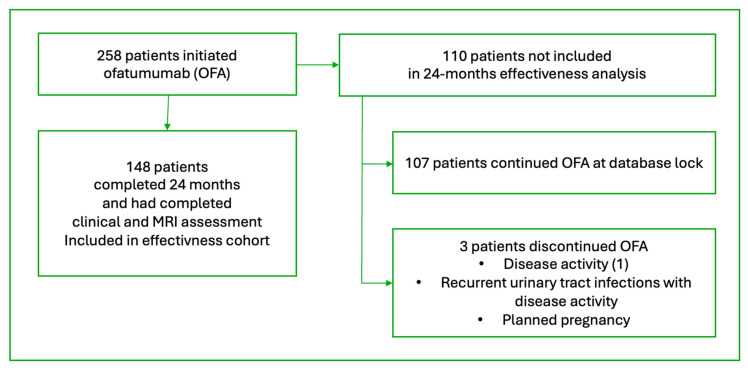
Study flowchart.

At database lock, 110 patients had not yet completed the annual assessment; 107 remained on OFA, while three discontinued therapies (Figure 1). Discontinuations were due to chronic urinary tract infections with disease activity (n = 1), disease activity alone (n = 1), and planned pregnancy (n = 1). Two occurred before 12 months and one between 12 and 24 months; these patients were excluded from the effectiveness analysis.

### 3.2. Clinical and Radiological Efficacy Outcomes

Within the first 12 months of treatment, relapses were documented in 20 patients (13.51%), new or enlarging T2-W lesions in 20 patients (13.51%), and GELs in seven patients (4.73%). EDSS progression was reported in 15 patients (10.14%), while EDSS improvement was observed in 20 patients (13.51%). NEDA-3 was achieved in 116 patients (78.37%) during the observation period (Table 2).

During the subsequent 12 months of treatment, relapses were documented in four patients (2.70%), new or enlarging T2-W lesions in three patients (2.03%), and GELs in zero patients (0%). EDSS progression was reported in 12 patients (8.10%), while EDSS improvement was observed in five patients (3.37%). NEDA-3 was achieved in 133 patients (89.86%) during this period (Table 2).

Over the entire 24-month treatment period, relapses were documented in 23 patients (15.54%), new or enlarging T2-W lesions in 23 patients (15.54%), and GELs in seven patients (4.73%). EDSS progression was reported in 26 patients (17.56%); one patient experienced progression in both the first and the second year of therapy. EDSS improvement was observed in 25 patients (16.89%). NEDA-3 was achieved in 106 patients (71.62%) during the observation period (Table 2).

In the unadjusted comparisons shown in Table 2, no statistically significant differences were detected between treatment-naïve and switched groups at 12 months, 12–24 months, or over the full 24-month period.

Across the first 12 months of therapy and the subsequent 12 months, a significant reduction in relapse frequency and MRI disease activity was observed compared with the 12 months prior to OFA initiation (Table 3).

### 3.3. Safety Assessment

In the mature-follow-up safety subgroup, 42/148 patients (28.38%) experienced at least one recorded AE, with similar rates in the switched and treatment-naïve groups as presented in Table 4. Most AEs were mild, and no serious adverse events (SAEs) were reported. Infections occurred in 39/148 patients (26.35%), again with comparable frequencies between switchers and naïve patients (Table 4). Respiratory tract infections were most common (34 patients, 22.97%), followed by urinary tract infections (12 patients, 8.10%), herpes simplex virus infections (12 patients, 8.10%), and gastrointestinal infections (5 patients, 3.38%). All infections were graded according to the Common Terminology Criteria for Adverse Events (CTCAE); all were grade 1–2, and no grade ≥3 infections, hospitalizations, or SAEs occurred. Injection-related reactions (IRRs) were reported in 71 of 148 patients (48.0%), with no significant difference between switched and treatment-naïve patients (Table 4).

### 3.4. Baseline Factors Associated with NEDA-3 Non-Achievement After 24 Months of OFA

An exploratory multivariable logistic regression model was used to assess baseline factors associated with failure to achieve NEDA-3 after 24 months of OFA treatment in the effectiveness cohort. Covariates included age, sex, disease duration, treatment status at OFA initiation (treatment-naïve vs. previously treated), prior exposure to HET before OFA, and baseline EDSS. Baseline EDSS at OFA initiation was a significant predictor of not achieving NEDA-3 (Table 5). The logistic regression model demonstrated moderate discriminatory ability, with an AUC of 0.75 (95% CI: 0.66–0.84).

## 4. Discussion

In this 24-month real-world cohort from tertiary MS centers, OFA was associated with sustained suppression of inflammatory activity in RRMS. Compared with the 12 months preceding treatment initiation, both clinical and radiological activity were markedly reduced during the first year of therapy and remained low during 12–24 months. These findings extend our previous 12-month analysis and suggest that early disease control achieved with OFA may be maintained over a two-year period in routine clinical practice.

Interval NEDA-3 was high in year 1 and higher in year 2; cumulative 24-month NEDA-3 was lower, reflecting the stringent nature of this composite endpoint and the impact of early events on long-term NEDA-3 status. These findings extend our prior 12-month multicenter analysis, showing that early disease control can be maintained for two years within a reimbursed national program, with particularly low clinical and radiological activity in year 2. Findings are consistent with ASCLEPIOS I/II [5] and ALITHIOS [6]. Higher NEDA-3 rates in routine practice may reflect baseline features, local eligibility criteria, and monitoring intensity (annual MRI vs. more frequent trial imaging), as well as our cohort’s low baseline disability.

We also did not detect statistically significant differences between treatment-naïve and switched patients in unadjusted comparisons, but this should not be interpreted as equivalence because the groups differed at baseline, particularly in age and disease duration.

A growing body of real-world evidence suggests that our 24-month OFA findings fall within the range expected for anti-CD20 therapy in routine practice rather than clearly exceeding it. In the Italian multicenter study by Ferrazzano et al., ARR decreased from 0.9 to 0.02, EDSS remained stable, and no MRI activity was detected at 24 months, although only 103 patients had complete 24-month follow-up and the mean follow-up across the cohort was 15.4 months [10]. In Croatia, a larger multicenter cohort of 665 patients, monitored every six months, showed a reduction in ARR from 0.70 to 0.03 and NEDA-3 in 76.7% of patients over a median follow-up of 1.55 years [11]. Additional OFA studies from the United States and Sicily likewise reported marked relapse suppression, high short-term persistence, and broadly comparable outcomes in treatment-naïve and switched patients [12,13], whereas German patient-centered data emphasized good tolerability and improved subjective quality of life [14]. Additionally, a recent Polish multicenter retrospective study including 430 patients with RRMS treated with OFA found that 72.7% achieved NEDA-3 after the first treatment year [15]. Similarly, a large U.S. claims-based study of 779 patients reported a 75% reduction in ARR after OFA initiation (from 0.41 to 0.10), accompanied by significant decreases in MS-related hospitalizations, hospital days, and outpatient visits [16]. Importantly, however, these studies did not always evaluate the same endpoints; for example, the US claims-based analysis assessed relapse and healthcare utilization, but not MRI activity or formal NEDA-3.

At the same time, comparable inflammatory disease control has also been described in real-world cohorts treated with other anti-CD20 agents, such as ocrelizumab (OCR) and rituximab (RTX). In an Italian propensity-score matched study, OFA and OCR showed similar 12-month ARR and NEDA-3 rates (94.5% vs. 92.1%); however, that cohort excluded patients switched from prior high-efficacy therapies and underwent MRI at 6 and 12 months [17]. In the larger German prospective comparison by Meuth et al., patients were reviewed every 3 months and underwent semiannual MRI, and OFA was non-inferior to OCR with respect to relapses, new or enlarging MRI lesions, and disability worsening [18]. Real-world OCR cohorts have also reported 24-month NEDA-3 rates of 84.6% in two Swiss centers [19] and 84.9% in an Italian cohort [20], whereas the longer MSBase registry study observed a 4-year NEDA-3 probability of 39.7% in an older and more disabled population [21]. Rituximab studies likewise support strong anti-inflammatory effectiveness, with NEDA-3 achieved in 79% of a Norwegian cohort [22], although a registry-based comparison suggested a higher relapse risk with rituximab than with ocrelizumab in routine care [23]. 

These cross-study differences likely reflect not only the expected efficacy of OFA, but also important differences in cohort composition and monitoring strategy, including our cohort’s low baseline disability (median EDSS 1.5 in both treatment-naïve and switched patients), annual MRI surveillance within the Polish drug program, and restriction of the 24-month analysis to patients with complete clinical and MRI follow-up. Accordingly, our findings are best interpreted as showing durable OFA effectiveness in a selected national-program population and as broadly consistent with the expected effects of anti-CD20 therapy in routine practice, rather than as evidence of clear superiority over other published cohorts.

Safety over 24 months was favorable: AEs were mild, with no serious events; infections were CTCAE grade 1–2 only, with no grade ≥3, hospitalizations, or complications. Retrospective documentation may underreport minor AEs, and immunoglobulins were not systematically assessed. IRRs were common early but waned. A Sicilian prospective cohort (n = 213) showed similar 12-month tolerability [13]; FDA Adverse Event Reporting System (FAERS) reports mainly infections and injection/systemic reactions, supporting ongoing monitoring [24].

Baseline EDSS was the only factor associated with NEDA-3 non-achievement in the exploratory regression analysis. This may suggest that greater pre-existing disability reflects a more treatment-resistant phenotype; however, the result should be interpreted cautiously because the number of events was limited and clinically relevant confounders, such as baseline MRI lesion burden, pretreatment relapse frequency, and time since last relapse, were not available in the present model. Therefore, the identified association between baseline EDSS and NEDA-3 non-achievement should be considered exploratory and hypothesis-generating rather than definitive.

This retrospective real-world study has several limitations. The effectiveness analysis was restricted to patients with complete 24-month follow-up and full clinical and MRI assessment, which introduces a potential risk of selection and survivorship bias. Patients who discontinue therapy early due to adverse events or insufficient treatment response are not represented in the complete-case cohort, which may lead to an overestimation of treatment effectiveness, particularly the NEDA-3 rate. Therefore, the findings are best interpreted as outcomes in a mature follow-up subcohort rather than as a global estimate of 24-month effectiveness in the full treated population. Additionally, AEs were collected retrospectively from routine records, which may underreport mild or transient events. In addition, immunoglobulin concentrations were not systematically available across centers, so laboratory abnormalities such as hypogammaglobulinemia may have gone unrecognized. Strengths include multicenter national-program recruitment, two-year follow-up extending the prior 12-month analysis to assess durability, and parallel evaluation of treatment-naïve and switched patients using clinically relevant endpoints.

## 5. Conclusions

In this multicenter retrospective real-world study, OFA was associated with low inflammatory disease activity over 24 months in RRMS patients with mature follow-up. Nevertheless, these results should be interpreted with caution because the 24-month effectiveness analysis was based on a complete-case subcohort, and safety data were collected retrospectively. Larger prospective studies with longer follow-up and more complete safety and laboratory capture are warranted.

## Figures and Tables

**Table 1:** Baseline characteristics of the 24-month effectiveness cohort (patients with mature follow-up and complete clinical and MRI assessment), according to treatment status at OFA initiation.

Characteristics	Patients Who Switched from Another DMT (N = 92)	Naïve Patients (N = 56)	*p*
Female/male, n (%)	65/27 (70.7/29.3%)	41/15 (73.2/26.8%)	0.73
Age, years, mean ± SD	39.27 ± 11.2	35.07 ± 9.53	0.02
Duration of disease, years, mean ± SD	11.44 ± 6.46	4.25 ± 4.32	<0.001
EDSS, median (min-max)	1.5 (1–4.5)	1.5 (0–4.5)	0.4

DMT—disease-modifying therapy, SD—standard deviation, EDSS—Expanded Disability Status Scale.

**Table 2:** Clinical and MRI efficacy outcomes after the first 12 months, during months 12–24, and over the entire 24-month period of ofatumumab treatment in patients switched from another DMT versus treatment-naïve patients.

	First 12 Months of Ofatumumab Treatment			Second 12 Months of Ofatumumab Treatment (Months 12–24)			24 Months of Ofatumumab Treatment		
Efficacy of Treatment Parameters	Switched from Another DMT (N = 92)	Treatment -Naïve (N = 56)	*p* (χ^2^)	Switched from Another DMT (N = 92)	Treatment -Naïve (N = 56)	*p* (χ^2^)	Switched from Another DMT (N = 92)	Treatment -Naïve (N = 56)	*p* (χ^2^)
Relapses, n (%)	13 (14.13)	7 (12.50)	0.77	3 (3.26)	1 (1.79)	1.00	15 (16.3)	8 (14.29)	0.74
T2-W lesions, n (%)	13 (14.13)	7 (12.50)	0.77	3 (3.26)	0	0.28	16 (17.39)	7 (12.50)	0.43
GELs, n (%)	5 (5.43)	2 (3.57)	0.71	0	0	1.00	5 (5.43)	2 (3.57)	0.71
EDSS progression, n (%)	10 (10.87)	5 (8.92)	0.79	8 (8.69)	4 (7.14)	1.00	17 (18.47)	9 (16.07)	0.71
EDSS improvement, n (%)	12 (13.04)	8 (14.28)	0.83	1 (1.08)	4 (7.14)	0.07	13 (14.13)	12 (21.42)	0.25
NEDA-3, n (%)	70 (76.08)	46 (82.14)	0.39	83 (90.21)	50 (89.28)	0.85	64 (69.56)	42 (75.00)	0.47

DMT—disease-modifying therapy; T2-W—T2-weighted (lesions); GELs—gadolinium-enhancing lesions; EDSS—Expanded Disability Status Scale; NEDA-3—No Evidence of Disease Activity-3.

**Table 3:** Clinical and MRI activity during the 12 months before OFA initiation and during the first and second year of OFA treatment in patients switching from another DMT versus treatment-naïve patients.

Disease Activity	12 Months Before OFA	0–12 Months of OFA Treatment	12–24 Months of OFA Treatment	*p* *
All patients				
Relapses, n (%)	81 (54.73)	20 (13.51)	4 (2.70)	<0.001
MRI activity (T2-W and/or GEL), n (%)	65 (43.92)	21 (14.19)	3 (2.02)	<0.001
Treatment-naïve				
Relapses, n (%)	52 (92.85)	7 (12.50)	1 (1.79)	<0.001
MRI activity (T2-W and/or GEL), n (%)	24 (42.85)	7 (12.50)	0	<0.001
Switched group				
Relapses, n (%)	29 (31.52)	13 (14.13)	3 (3.26)	<0.001
MRI activity (T2-W and/or GEL), n (%)	41 (44.56)	14 (15.22)	3 (3.26)	<0.001

OFA—ofatumumab; MRI—magnetic resonance imaging; T2-W—T2-weighted lesions; GELs—gadolinium-enhancing lesions; * Cochran’s Q test, pairwise comparisons between intervals using McNemar’s test.

**Table 4:** Adverse events, infections, and injection-related reactions over 24 months of ofatumumab treatment in switched vs. treatment-naïve patients.

Events	Patients Who Switched from Another DMT (N = 92)	Treatment-Naïve Patients (N = 56)	*p* (χ^2^)
Adverse events, n (%)	25 (27.18)	17 (30.36)	0.68
Infections, n (%)	24 (26.09)	15 (26.79)	0.93
Injection-related reactions, n (%)	44 (47.82)	27 (48.21)	0.96

DMT—disease-modifying therapy.

**Table 5:** Multivariable logistic regression analysis of baseline predictors of not achieving NEDA-3 after 24 months of ofatumumab treatment.

Predictor	OR	95% CI	*p*-Value
Age (per year)	1.10	0.96–1.94	0.94
Male sex	1.11	0.47–2.61	0.81
Disease duration (per year)	0.98	0.92–1.08	0.95
Treatment-naïve (vs switched)	1.18	0.45–3.03	0.73
HET before OFA	0.60	0.18–1.97	0.39
Baseline EDSS (per point)	1.73	1.05–2.84	0.03

CI—confidence interval; EDSS—Expanded Disability Status Scale; HET—high-efficacy therapy; NEDA-3—No Evidence of Disease Activity-3; OFA—ofatumumab; OR—odds ratio.

## Data Availability

The data presented in this study are not publicly available due to privacy restrictions.

## Abbreviations

The following abbreviations are used in this manuscript:

AE(s)	Adverse event(s)
ARR	Annualized relapse rate
AUC	Area under the curve
CD20	Cluster of differentiation 20
CI	Confidence interval
CTCAE	Common Terminology Criteria for Adverse Events
DMT(s)	Disease-modifying therapy/therapies
EDSS	Expanded Disability Status Scale
FAERS	FDA Adverse Event Reporting System
FDA	(U.S.) Food and Drug Administration
GEL(s)	Gadolinium-enhancing lesion(s)
HET	High-efficacy therapy/therapies
IQR	Interquartile range
IRR(s)	Injection-related reaction(s)
MRI	Magnetic resonance imaging
MS	Multiple sclerosis
N/n	Number (sample size/count)
NEDA-3	No Evidence of Disease Activity-3
OCR	Ocrelizumab
OFA	Ofatumumab
OR	Odds ratio
RRMS	Relapsing–remitting multiple sclerosis
RTX	Rituximab
RWE	Real-world evidence
SAE(s)	Serious adverse event(s)
SD	Standard deviation
T2-W	T2-weighted (lesions)
